# The Availability of Improved Sanitation Facilities and Its Associated Factors in the 12^th^ District of Kandahar City, Afghanistan

**DOI:** 10.1155/2021/5569582

**Published:** 2021-09-03

**Authors:** Esmat Ullah Muslim, Muhammad Haroon Stanikzai, Abdul Wahed Wasiq, Ahmad Khan, Hadia Sayam

**Affiliations:** ^1^Master of Public Health Program, Kandahar University, Kandahar, Afghanistan; ^2^Public Health Department, Faculty of Medicine, Kandahar University, Kandahar, Afghanistan; ^3^Internal Medicine Department, Faculty of Medicine, Kandahar University, Kandahar, Afghanistan; ^4^Clinic Department, Faculty of Medicine, Mirwais Neka Institute of Higher Education, Kandahar, Afghanistan; ^5^Para-Clinic Department, Faculty of Medicine, Malalay Institute of Higher Education, Kandahar, Afghanistan

## Abstract

**Background:**

The majority of people practicing open defecation and utilizing unhealthy sanitation facilities are in the developing world. The utilization of unimproved sanitation facilities remains the primary risk factor for many diseases, including nutritional diseases, diarrheal diseases, typhoid, cholera, and dysentery, particularly among children.

**Objectives:**

This study was carried out to assess the availability of improved sanitation facilities and factors associated with it in the 12^th^ district of Kandahar city, Kandahar Province, Afghanistan.

**Methods:**

The study is a cross-sectional survey, conducted between September and October 2019. A structured questionnaire was used to gather self-reported information of the respondents, including sociodemographic information, household characteristics, and behavioral and environmental characteristics of the available sanitation facilities. Factors associated with the availability of the improved sanitation facility were determined using a multivariable logistic regression model.

**Results:**

In this study, the availability of improved sanitation facilities was 85.7% (95% confidence interval (CI) = 77.6%–92.1%). It was significantly influenced by living in a private house (adjusted odds ratio (AOR) = 2.99 (95% CI; 1.43–6.26)); inside location of latrine (AOR = 14.31 (95% CI; 3.59–56.99)); individual household latrine (AOR = 2.03 (1.04–3.95)); and the number of latrines in the household (AOR = 5.04 (2.45–10.35)).

**Conclusion:**

The availability of improved sanitation facilities was higher compared to the national level in the study area. This study provides significant evidence on approaches in line with the World Health Organization's (WHO) Joint Monitoring Program and Sustainable Developmental Goals (SDGs) for enhancing the availability of improved sanitation facilities in Kandahar city.

## 1. Introduction

Ensuring access to improved sanitation facilities is an increasing challenge for many low-income countries. The majority of people practicing open defecation and those utilizing unhealthy sanitation facilities are in the developing world [1]. The utilization of unimproved sanitation facilities remains the primary risk factor for many diseases, including nutritional diseases, diarrheal diseases, typhoid, cholera, and dysentery, particularly among children [2–4].

The World Health Organization's (WHO) Joint Monitoring Program (JMP) defines improved sanitation facilities as “a sanitation system in which excreta are disposed of in such a way that they reduce the risk of fecal-oral transmission to its users and the environment” and includes “flush or pour-flush to a piped sewer system, septic tank or pit latrine, ventilated improved pit latrine, pit latrine with slab, and composting toilet” [5].

The WHO has estimated that nearly two billion individuals are utilizing unhealthy sanitation facilities, 673 million of whom are practicing open defecation [1, 5]. In Afghanistan, only 25% of individuals have access to improved sanitation facilities. It is also evident from the Afghanistan Demographic Health Survey (ADHS, 2015) that urban areas are more (32%) likely to own an improved sanitation facility [6].

Different studies in developing countries have identified that household wealth status [7–11], residence (urban/rural) [7, 8], household head's characteristics (age, gender, level of education, and employment status) [7, 8, 10, 11], and cultural [8–10] and religious beliefs [11, 12] are significantly associated with the availability of improved sanitation facilities.

One of the key targets in Sustainable Development Goals (SDGs) 2 is as follows: “By 2030, achieve access to adequate and equitable sanitation and hygiene for all and end open defecation, paying special attention to the needs of women and girls and those in vulnerable situations” [13]. To achieve this target, data on the factors (individual, household, and system-related factors) associated with the availability of improved sanitation facilities are needed to promote healthy living in Afghanistan. Hence, our objective in this study was to assess the availability of improved sanitation facilities and factors associated with it in the 12^th^ district of Kandahar city. The population in the present study can be representing a typical urban district from the south of Afghanistan in terms of socioeconomic and cultural characteristics. Therefore, the findings of this study will help to design evidence-based policies to enhance the availability of improved sanitation facilities across southern Afghanistan.

## 2. Materials and Methods

### 2.1. Study Setting and Design

This study was a community-based cross-sectional survey of randomly selected households in the 12^th^ district of Kandahar city, which was conducted between September and October 2019. Besides its original residents, this district has been home to many Internally Displaced Persons (IDPs). It is the largest district with approximately 90000 people and ten villages, some 10 km north of the central zone. Two schools and one comprehensive health clinic are found in the district. Sketchy maps were available for all villages, and all households within the villages were numbered.

### 2.2. Sample Size and Sampling Procedure

The sample size was calculated based on the single population proportion formula [14]; considering the assumption of 95% confidence interval and 5% margin of error, *p*=0.5 is the estimate for the proportion of households with an improved sanitation facility (since there was no study). Allowing for a 10% nonresponse rate, a sample size of 450 was adequate.

A stratified systematic random sampling method was used to select a sample of 50 households per village. In each village, the sampling interval (*k*) was determined as the ratio of households in the village to sample size. We used a random number from 1 to *k* to select a starting household, and afterward, every *k*th household was included in the study.

We enrolled all households in the 12^th^ district of Kandahar city. Households not available during the study period or who declined to participate were excluded.

### 2.3. Data Collection

At each household, the head or other adult members of the household were interviewed. Informed verbal consent was obtained from all respondents. The study instruments were initially prepared in English and translated to Pashtu and back to English to ensure the meaning of the questions was preserved during translation. It was pilot tested on 5% of the total sample in another setting (Aino Mena, Kandahar city) before starting the study. The questionnaire gathered self-reported information of the respondents, including sociodemographic characteristics, household characteristics, and behavioral and environmental characteristics of the sanitation facility available.

The data was collected by three pairs of local interviewers (one male and one female) and one supervisor (health professional). Before the pilot study, the principal investigators provided a two-day training session to the data collectors. It was focused on sampling methods, interview techniques, filling out questionnaires, and ethical issues during the study. The principal investigators monitored the data collection through random surveys of households. The questionnaires were checked for completion and quality daily.

### 2.4. Statistical Analysis

All questionnaires were first coded and entered into Microsoft Excel (2019) and later exported into Statistical Package for Social Sciences (SPSS) version 21 for data cleaning and analysis [15]. The availability of improved sanitation facilities was calculated at the household level. A binary logistic regression model was used to assess factors associated with the availability of improved sanitation facilities. Variables with *p* value of less than 0.25 were retained in multivariable logistic regression. Finally, a multivariable logistics analysis was carried out to determine independent predictors of improved sanitation facility availability. *p* value of <0.05 was considered statistically significant.

### 2.5. Ethical Consideration

This community-based study received ethical clearance from the Research and Ethics Committee of Kandahar University (Maktob No. 53, Date: 28/7/2019). Administrative approval was obtained from the Kandahar municipality to conduct this study.

## 3. Results

### 3.1. Sociodemographic Characteristics of the Respondents

In this study, a total of 450 households, representing 6052 persons, were included. Of all respondents, 439 (97.6%) were male. The mean age and standard deviation of the respondents were 29.49 ± 7.2. Of the total, about 62.7% (282) were within the age range of 21–30 years. Two hundred ninety (64%) of the respondents were married, and the remaining 35.6% (160) were single. About one-third (32%) of the respondents had secondary education, whereas 120 (26.7%) had no formal education. The majority (98.2%) of the respondents were employed. The average household size was 13.45 persons. An 80.4% (362) of the household size was within the range of 11–20 persons. About 79.1% (356) of the households had an average monthly income in the range of 5000–10000 Afghanis (100–150 USD) while 94 (20.9%) households had an average monthly income range of >10000 Afghanis (>150 USD) (Table 1).

### 3.2. House, Water, and Sanitation Facility Characteristics

Of the households included in the study, about 62% (282) of the houses were constructed with mud, and the majority 89.6% (403) of the households were male-headed. The time since the household has been living in this house compound was one year or more for 365 (81.1%) respondents. Most houses (93.6%) had electricity and all houses (100%) had access to sanitation facilities. More than half (58.2%) of the households used hand pumps, while 105 (23.3%) used water tanks as a water source. The majority (94%) of latrines were located inside the house compound and about 43.3% (195) shared their sanitation facilities with other households. Around half (51.3%) received a subsidy in constructing the latrine (Table 2).

**Figure 1:**
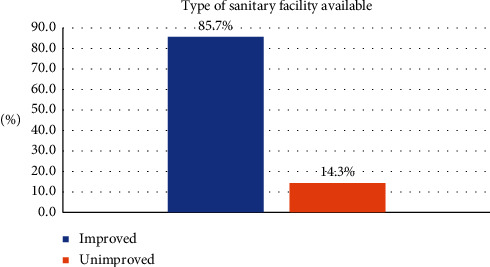
The proportion of improved sanitation facilities available in the 12^th^ district of Kandahar city, 2019.

In this study, 386 (87.5%, 95% confidence interval (CI); 77.6%–92.1%) households had an improved sanitation facility (Figure 1), 45 (10%) were using pit latrine without a slab, 13 (2.9%) were using hanging latrines, and only 6 (1.6%) were practicing open defecation. More than half (57.1%) of the households put their children's feces into the latrine (Table 2).

### 3.3. Behavioral, Environmental, and Other Medical-Related Characteristics

Of all respondents, about 85.6% (385) reported cleaning their latrines. Of these 385 respondents, 35.8% (161) were cleaning the latrine rarely, while 124 (27.6%) reported cleaning once a week. The majority (88.9%) of the respondents stated that they utilized household latrines the last time they were defecating. Of them, most (84.4%) of the respondents declared that they washed their hands after defecation. Major reasons for not utilizing latrines were latrine dysfunctionality (32%), lack of privacy (24%), and hygienic issues (14%). About 45.1% of the respondents reported that there was a positive diarrhea case in the past week, while nearly one-third of the respondents stated that there was a positive case of malnutrition in the past 3 months in the household. The detailed characteristics of the respondent's behavioral, environmental, and other medical-related characteristics are shown in Table 3.

### 3.4. Factors Associated with the Availability of Improved Sanitation Facilities

Variables that were significantly associated with the availability of improved sanitation facilities in the bivariate analysis included respondent's educational status, living in a private house, inside location of the latrine, individual household latrine, latrine cleaning, hand washing after defecation, latrine distance from the water source, and the number of latrines. The factors that remained significantly associated with the availability of improved sanitation facilities in multivariate analysis were living in a private house, with adjusted odds ratio (AOR) = 2.99 (95% CI; 1.43–6.26); inside location of latrine, with AOR = 14.31 (95% CI; 3.59–56.99), individual household latrine, with AOR = 2.03 (95% CI; 1.04–3.95); and the number of latrines, with AOR = 5.04 (95% CI; 2.45–10.35) (Table 4).

## 4. Discussion

This study assesses the availability of improved sanitation facilities and their associated factors in the 12^th^ district of Kandahar city. In this study, we found that 85.7% of households had at least one improved sanitation facility. Additionally, we found factors such as living in a private house, inside location of the latrine, two or more latrines, and individual household latrines were associated with the availability of the improved sanitation facility.

The availability of improved sanitation facilities (85.7%) in the present study is higher compared to national reports and other studies from Afghanistan [6, 16, 17]. However, this rate is lower than the one reported in studies from India [12], Bangladesh [18], Pakistan [19], and Ethiopia [20]. This variation reported in the proportion of improved sanitation facilities availability in studies may be explained by variation in socioeconomic status, cultural differences, geographical location, research design, religious beliefs, and other unexplored factors.

This study found that 38% of the households lived in their own houses. As to the factors associated with the availability of the improved sanitation facility, households living in their private house were about three times more likely to have an improved sanitation facility compared to those living in a rented or leased house. The lower rate of improved sanitation facilities among those living in a rented or leased house may partly be explained by the low socioeconomic status of the dwelling households, making it difficult for them to construct an improved sanitation facility [8–10]. As the economic situation of these households remains fragile, identifying means to provide subsidies at an affordable cost is of grave importance.

In this study, the majority (94%) of the latrines were constructed inside the household compound. Furthermore, houses with an inside latrine were 14.13 times more likely to have an improved sanitation facility. Studies show that most cases where latrines are constructed inside the compound also influence latrine utilization [21, 22]. Looking at this scientific evidence, it is imperative that program implementers should acknowledge households to construct a latrine inside the compound.

This study revealed that only 56.7% (255) households owned a sanitary facility which was not shared with other households. We further observed that households with their own latrine had 2.03 times the odds of having an improved sanitation facility. It is widely believed that shared sanitation facilities are unacceptable in terms of both accessibility and cleanliness [5, 23, 24]. Furthermore, shared sanitation facilities are more likely to cause psychosocial stress due to a lack of privacy and safety, particularly among women [25–28].

Little is known about the relationship between the number of latrines and the availability of improved sanitation facilities. Previous literature revealed that households with better socioeconomic status are more likely to construct more and improved sanitation facilities [9–12]. Our study found that households with two or more latrines were about five times more likely to have an improved sanitation facility.

Although other sociodemographic factors such as age, educational status, employment, and wealth were identified as significant predictors for the availability of improved sanitation facilities in the literature, albeit inconsistent [7–12], this present study has identified no association. This may indicate possible differences in sociodemographic characteristics of the study participants.

This study found the proportion of improved sanitation facilities and their associated factors in the 12^th^ district of Kandahar city. The findings of this study, however, should be considered in light of its limitations. The cross-sectional nature of the study limits the temporal relationship between variables. Moreover, there will be information bias as the respondents self-reported the type of sanitation facility available in the house compound that can be over or underreported. Concerning the sampling procedure, sketchy maps may have resulted in bias, particularly if new houses were built. Lastly, the study involved only one district that merely limits its generalizability.

## 5. Conclusion

The availability of improved sanitation facilities was higher compared to the national level in the study area. The availability of the improved sanitation facilities was influenced by the ownership of the house, the inside location of the latrine, latrines not shared with other households, and the number of latrines. Identifying means to provide subsidies at an affordable cost and constructing latrines inside house compounds is critical for the availability of improved sanitation facilities.

## Figures and Tables

**Table 1:** Sociodemographic characteristics of respondents in the 12^th^ district of Kandahar city, 2019 (*n* = 450).

Variables	Frequency (%)
*Age groups*	
15–20	20 (4.4)
21–30	282 (62.7)
31–40	116 (25.8)
41–50	27 (6.0)
51–60	5 (1.1)

*Gender*	
Male	439 (97.6)
Female	11 (2.4)

*Marital status*	
Single	160 (35.6)
Married	290 (64.4)

*Educational level*	
No formal education	120 (26.7)
Religious (madrasa)	82 (18.2)
Primary	18 (4.0)
Secondary	144 (32.0)
Higher education	86 (19.1)

*Occupation*	
Government	96 (21.3)
Private	117 (26.0)
Self-employed	229 (50.9)
Unemployed	8 (1.8)

*Language spoken*	
Pashtu	362 (80.4)
Dari	88 (19.6)

*Members of household*	
1–10	76 (16.9)
11–20	362 (80.4)
21–30	12 (2.7)

*Household monthly income (in Afghanis)*	
5000–10000	356 (79.1)
>10000	94 (20.9)

**Table 2:** House, water, and sanitation facility characteristics in the 12^th^ district of Kandahar city, 2019 (*n* = 450).

Variables	Frequency (%)
*Type of house*	
Cement	20 (4.4)
Mud and clay	282 (62.7)
Mix	128 (28.4)

*Ownership of house*	
Private	171 (38.0)
By lease	159 (35.3)
Others	120 (26.7)

*Length of time living*	
<1 year	85 (18.9)
≥1 year	365 (81.1)

*Electricity*	
Yes	421 (93.6)
No	29 (6.4)

*Water source*	
Hand pump	262 (58.2)
Water tank	105 (23.3)
Dug well	42 (9.3)
Filter water	18 (4.0)
Water pipe	18 (4.0)
Steam/well	5 (1.1)

*Household head*	
Male	403 (89.6)
Female	47 (10.4)

*Latrine available*	
Yes	450 (100.0)
No	0 (0)

*Number of latrines available*	
One	183 (40.7)
Two	201 (44.7)
Three	61 (13.6)
Four	5 (1.1)

*Latrine location*	
Inside	423 (94.0)
Outside	27 (6.0)

*Type of sanitation facility (adults)*	
Flush to septic tank	21 (4.7)
Flush/pour to pit latrine	55 (12.2)
Pit latrine with slab	129 (28.7)
Ventilated pit latrine	181 (40.2)
Pit latrine without slab	45 (10.0)
Hanging toilet	13 (2.9)
Open defecation	6 (1.3)

*Shared with other households*	
Yes	195 (43.3)
No	255 (56.7)

*Distance of latrine from water source*	
>10 m	323 (71.8)
<10 m	127 (28.2)

*Received any subsidy in constructing the latrine*	
Yes	231 (51.3)
No	219 (48.7)

*Type of defecating facility (children)*	
Put into latrine	257 (57.1)
Use latrine	111 (24.7)
Put into drain or ditch	24 (5.3)
Buried	44 (9.8)
Thrown into garbage	8 (1.8)
Open defecation	6 (1.3)

**Table 3:** Behavioral, environmental, and other medical-related characteristics.

Variables	Frequency (%)
*Latrine cleaning (n* = 450)	
Yes	385 (85.6)
No	65 (14.4)

*Frequency of latrine cleaning (n* = 385)	
Every day	38 (8.4)
2-3 times a week	62 (13.8)
Once a week	124 (27.6)
Rarely	161 (35.8)

*Method of cleaning (n* = 385)	
Water	237 (52.7)
Water + soap	86 (19.1)
Bleach	36 (8.0)
Mixed methods	26 (5.8)

*Use of latrine the last time defecated (n* = 450)	
Yes	400 (88.9)
No	50 (11.1)

*Reasons for not using latrine (n* = 50)	
Dysfunctional	16 (32)
Lack of privacy	12 (24)
Dirty	7 (14)
Dark (no light)	8 (16)
Prefer open defecation	5 (10)
Do not know	2 (4)

*Washed hands after defecation (n* = 400)	
Yes	380 (84.4)
No	20 (4.4)

*Diarrhea in the household in the past week (n* = 450)	
Yes	203 (45.1)
No	87 (19.3)
Not sure	160 (35.6)

*Child malnutrition in the household in the past* 3 *months (n* = 450)	
Yes	123 (27.3)
No	116 (25.8)
Not sure	211 (46.9)

**Table 4:** Factors associated with the availability of improved sanitation facilities in 12^th^ district of Kandahar city, 2019, showing crude and adjusted odds ratio.

Independent variable	Categories	Availability of sanitation facility		Crude odds ratio (95% CI)	Adjusted odds ratio (95% CI)
		Improved	Unimproved		
Respondent's education	Educated	292	38	2.12 (1.22–3.68)	—
	Uneducated	94	26	1	—

House ownership	Private	156	12	3.03 (1.56–5.87)	2.99 (1.43–6.26)
	Rent or lease	227	52	1	1

Latrine location	Inside	372	51	6.77 (3.01–15.22)	14.31 (3.59–56.99)
	Outside	14	13	1	1

Individual household latrine	Yes	14	13	1.87 (1.09–3.20)	2.03 (1.04–3.95)
	No	372	51	1	1

Latrine cleaning	Yes	337	48	2.29 (1.20–4.34)	—
	No	49	16	1	—

Handwashing after defecation	Yes	328	52	3.39 (1.29–8.90)	—
	No	13	7	1	—

Distance of latrine from water sources	≥10 meters	285	38	1.93 (1.11–3.34)	—
	<10 meters	101	26	1	—

Number of latrines	More than one	250	17	5.08 (2.81–9.19)	5.04 (2.45–10.35)
	One	136	47	1	1

## Data Availability

The primary data used to support the findings of this study are available from the corresponding author upon request.

## References

[1] World Health Organization (WHO) (2015). *Progress on Sanitation and Drinking Water—2015 Update and MDG Assessment*.

[2] Prüss‐Ustün A., Bartram J., Clasen T. (2014). Burden of disease from inadequate water, sanitation and hygiene in low‐and middle‐income settings: a retrospective analysis of data from 145 countries. *Tropical Medicine & International Health*.

[3] Bain R., Cronk R., Wright J., Yang H., Slaymaker T., Bartram J. (2014). Fecal contamination of drinking-water in low-and middle-income countries: a systematic review and meta-analysis. *PLoS Medicine*.

[4] Bartram J., Cairncross S. (2010). Hygiene, sanitation, and water: forgotten foundations of health. *PLoS Medicine*.

[5] WHO/UNICEF (2020). *The WHO/UNICEF Joint Monitoring Programme Estimates on WASH*.

[6] Central Statistics Organization (2017). *Afghanistan Demographic and Health Survey 2015*.

[7] Coffey D., Spears D., Vyas S. (2017). Switching to sanitation: understanding latrine adoption in a representative panel of rural Indian households. *Social Science & Medicine*.

[8] Slekiene J., Mosler H.-J. (2018). Characterizing the last latrine nonowners in rural Malawi. *The American Journal of Tropical Medicine and Hygiene*.

[9] Harter M., Mosch S., Mosler H.-J. (2018). How does community-led total sanitation (CLTS) affect latrine ownership? a quantitative case study from Mozambique. *BMC Public Health*.

[10] Alemu F., Kumie A., Medhin G., Gebre T., Godfrey P. (2017). A socio-ecological analysis of barriers to the adoption, sustainablity and consistent use of sanitation facilities in rural Ethiopia. *BMC Public Health*.

[11] Shakya H. B., Christakis N. A., Fowler J. H. (2015). Social network predictors of latrine ownership. *Social Science & Medicine*.

[12] Kaur R., Kant S., Lohiya A., Ahamed F., Malhotra S., Haldar P. (2020). Access and utilization of sanitation facilities in a rural area of Haryana, north India. *Indian Journal of Public Health*.

[13] SDG Indicators (2015). *Global SDG Indicators Database*.

[14] Bujang M. A. (2021). A step-by-step process on sample size determination for medical research. *Malaysian Journal of Medical Sciences*.

[15] International Business Machines Corporation (2012). *IBM SPSS Statistics for Windows, Version 21.0*.

[16] Mubarak M. Y., Wagner A. L., Asami M., Carlson B. F., Boulton M. L. (2016). Hygienic practices and diarrheal illness among persons living in at-risk settings in Kabul, Afghanistan: a cross-sectional study. *BMC Infectious Diseases*.

[17] Gon G., Monzon-Llamas L., Benova L., Willey B., Campbell O. M. R. (2014). The contribution of unimproved water and toilet facilities to pregnancy-related mortality in Afghanistan: analysis of the Afghan mortality survey. *Tropical Medicine & International Health*.

[18] Nelson K. B., Karver J., Kullman C., Graham J. P. (2014). User perceptions of shared sanitation among rural households in Indonesia and Bangladesh. *PLoS One*.

[19] Ghazanfar H., Saleem S., Naseem S., Ghazanfar A., Khattak U. K. (2017). Safe drinking water and sanitary measures: a cross-sectional study in peri-urban community of Islamabad. *JPMA. The Journal of the Pakistan Medical Association*.

[20] Gebremedhin G., Tetemke D., Gebremedhin M. (2018). Factors associated with latrine utilization among model and non-model families in Laelai Maichew Woreda, Aksum, Tigray, Ethiopia: comparative community based study. *BMC Research Notes*.

[21] Ajemu K. F., Desta A. A., Berhe A. A., Woldegebriel A. G., Bezabih N. M. (2020). Latrine ownership and its determinants in rural villages of Tigray, northern Ethiopia: community-based cross-sectional study. *Journal of Environmental and Public Health*.

[22] Yimam Y. T., Gelaye K. A., Chercos D. H. (2014). Latrine utilization and associated factors among people living in rural areas of Denbia district, Northwest Ethiopia, 2013, a cross-sectional study. *Pan African Medical Journal*.

[23] Heijnen M., Cumming O., Peletz R. (2014). Shared sanitation versus individual household latrines: a systematic review of health outcomes. *PLoS One*.

[24] Mara D. (2016). Shared sanitation: to include or to exclude?. *Transactions of the Royal Society of Tropical Medicine and Hygiene*.

[25] Shiras T., Cumming O., Brown J., Muneme B., Nala R., Dreibelbis R. (2018). Shared latrines in Maputo, Mozambique: exploring emotional well-being and psychosocial stress. *BMC International Health and Human Rights*.

[26] Saheem M., Stanikzai M. H., Rahimy N., Fazli N., Mudasir G. M., Sayam H. (2021). Factors associated with modern contraceptive use among married women attending comprehensive health centers (CHCs) in Kandahar, Afghanistan. *International Journal of Reproductive Medicine*.

[27] Stanikzai M. H., Wafa M. H., Wasiq A. W., Sayam H. (2021). Magnitude and determinants of antenatal care utilization in Kandahar city, Afghanistan.. *Obstetrics and Gynecology International*.

[28] Heijnen M., Routray P., Torondel B., Clasen T. (2015). Neighbour-shared versus communal latrines in urban slums: a cross-sectional study in Orissa, India exploring household demographics, accessibility, privacy, use and cleanliness. *Transactions of the Royal Society of Tropical Medicine and Hygiene*.

